# Obituary: Masatoshi Nei (1931–2023)

**DOI:** 10.1093/molbev/msad149

**Published:** 2023-06-28

**Authors:** Sudhir Kumar, Takashi Gojobori

**Affiliations:** Institute for Genomics and Evolutionary Medicine, Temple University, Philadelphia, PA, USA; Department of Biology, Temple University, Philadelphia, PA, USA; Marine Open Innovation Institute, Shizuoka-City, Japan; King Abdullah University of Science and Technology, Thuwal, Saudi Arabia

*Geneticist who Pioneered Evolutionary Methods, Championed Mutation-Driven Evolution, and Co-Founded MBE*

Masatoshi Nei, affectionately called Dr Nei by a legion of students and associates, was a brilliant geneticist renowned for his groundbreaking statistical studies that revolutionized our understanding of evolution at the molecular level. He pioneered numerous measures of genetic distance between populations and between sequences. Through the application of statistical methods, he led classic studies on overdominant selection in immune system genes, the discordance between gene trees and the species tree, and the evolutionary relationships of human populations. Dr Nei’s theory of mutation-driven evolution proposes mutation as the primary driver of evolutionary change, as nature cannot select without variation.

Born on January 2, 1931, in the Miyazaki prefecture of Japan, Masatoshi hailed from a family known for producing Shōchū, a Japanese liquor. Impressed by his academic talent, school teachers convinced his father to send him to college, which led to his graduation with a B.S. degree from the University of Miyazaki in 1953. Masatoshi’s abilities and research interests became quickly apparent with the publication of his first article on the mathematics of plant breeding as an undergraduate student in 1953. Dr Nei’s interest in plant breeding can be traced to his upbringing on a farm, as his parents and grandparents were all farmers. This heritage likely explains his lifelong interest in biology and genetics.

Six years later, he obtained his doctoral degree from the prestigious Kyoto University. However, he was unsatisfied with his doctoral work on quantitative genetics to improve crop plants. The weak results of his experimental studies and statistical analyses made him question the significance of genetic variation on annual crop yields in the presence of large environmental fluctuations in nature. He resolved to avoid such work in the future unless the environmental conditions could be controlled, as in laboratory studies of model organisms.

**Fig. 1.**
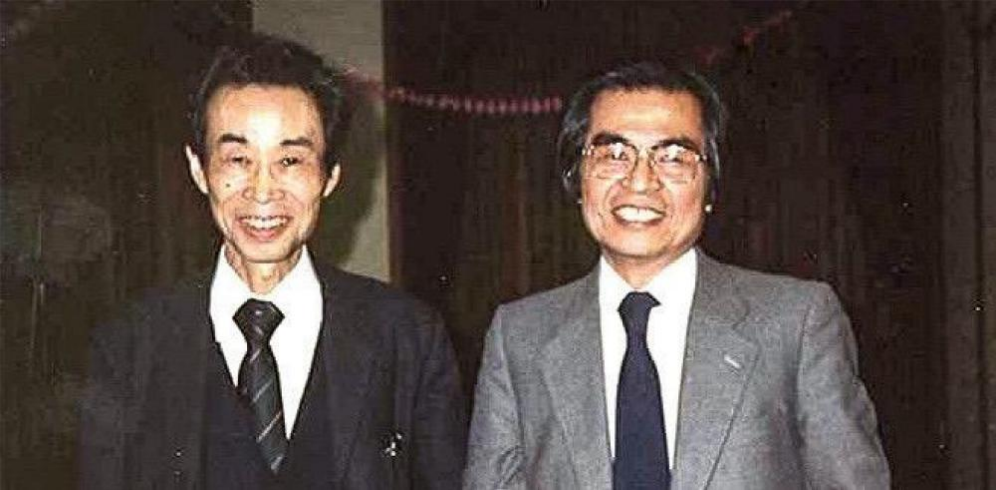
Masatoshi Nei (right) with Motoo Kimura (left) in a restaurant in 1986 in Mishima, Japan, home of the National Institute of Genetics. Photographer unknown; picture provided by Keitaro Nei.

In the early 1960s, after securing a position as a research scientist at the National Institute of Radiological Sciences in Japan, Dr Nei shifted his research focus from applied to basic science because many data sets on gene (allele) frequency became available. His interactions with Motoo Kimura at the National Institute of Genetics further accelerated this transition (fig. 1). Driven by data availability and greater molecular resolution, Dr Nei quickly modernized his research focus, subsequently analyzing restriction site polymorphisms and nucleotide sequences. Over his lifetime, his research program evolved from plant breeding to quantitative genetics, population genetics, molecular evolution, and eventually to molecular phylogenetics. This transformation was made possible by Dr Nei’s immigration to the United States in 1969, which was discouraged by Motoo Kimura, whom he considered his mentor.

During his time at Brown University in 1972, Dr Nei introduced a novel genetic distance measure, now known as Nei’s distance. This measure possessed the desirable property of equating to heterozygosity when comparing identical populations and scaling linearly with time. Dr Nei intentionally designed it to provide biologists with a unified measure to study population variation in terms of both genetic distance and heterozygosity. This became one of his early signature contributions. He also began distributing a computer program to calculate the new distance to help biologists apply it in their analyses. His approach of publishing new methods and providing accompanying computer programs became his modus operandi, now a standard practice in computational biology.

In the 1980s, Dr Nei delved into critical investigations of methods for constructing molecular phylogenies using synthetic data generated by computer simulations. Notably, he and his group members developed an interest in comparative analyses of nucleotide sequence data, as such data had just started to be published. However, it will surprise many to know that, in those days, even collecting the minimum three nucleotide sequences required to construct a phylogenetic tree necessitated inquiries at relevant universities and research institutions across the United States, an arduous and time-consuming task.

Dr Nei also published evolutionary relationships of human populations based on genetic data. In 1988, he coauthored a book with Arun Roychoudhury, titled “*Human Polymorphic Genes: World Distribution*,” which cataloged human gene frequency data. Around this time, Dr Nei’s research focus shifted toward evolutionary genetics and molecular evolution, as evidenced by the publication of his highly influential book “*Molecular Evolutionary Genetics*” in 1987, a decade after his first book on “Molecular Population Genetics and Evolution” in 1975. Dr Nei’s research emphasis was moving toward longer-term molecular evolution, driven by increased opportunities to make fundamental contributions. Despite the major resurgence of population genetics fueled by the invention of next-generation sequencing technologies in the 21st century, he did not return to population genetics studies.

**Fig. 2.**
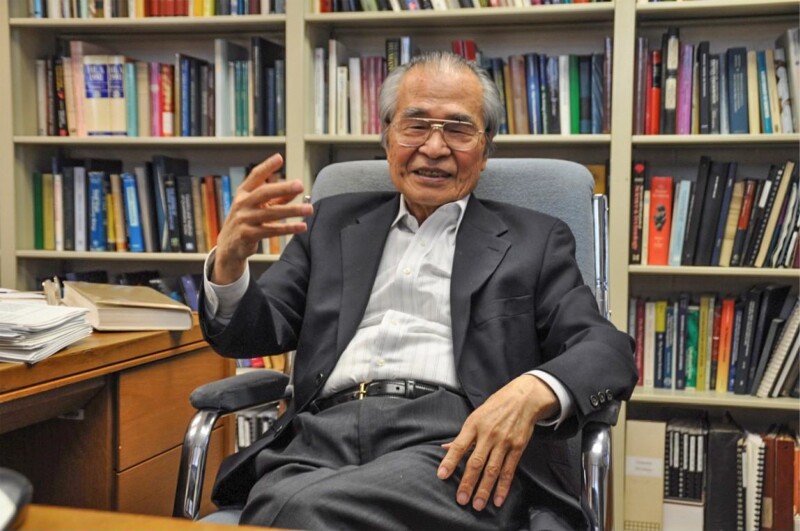
Masatoshi Nei at his office at Pennsylvania State University (year). Photographer unknown; picture provided by Keitaro Nei.

Collaborating with his students, Dr Nei published numerous statistical methods for analyzing molecular sequences. Biologists embraced these methods, resulting in numerous research articles citing his work. Notable contributions include the Nei–Gojobori (1986) method for estimating evolutionary distances based on nonsynonymous and synonymous substitutions, the Neighbor-Joining method (1987) and the minimum evolution method (1992) for reconstructing phylogenetic trees, and the Tamura-Nei (1993) model for calculating the number of nucleotide substitutions per site between sequences, and many others. Some of these methods were developed at the Pennslyvania State University, where he founded a new Institute for Molecular Evolutionary Genetics in 1990 (fig. 2).

Many of these statistical methods for comparative sequence analysis became more accessible with the release of the first version of the MEGA software in 1993. This integrated and user-friendly software replaced many piecemeal computer programs, becoming the next-generation product of Dr Nei’s laboratory to provide practical solutions to biologists. MEGA contained innovative statistical methods, algorithms, and a user-friendly interface, programmed by one of us (Sudhir Kumar) with Koichiro Tamura under Nei’s statistical guidance and financial support. Dr Nei’s administrative assistant, Joyce White, mailed over 2,500 copies of MEGA to interested users, each containing a computer diskette with the program and a comprehensive manual that provided instructions on program usage, methods, and algorithms. Dr Nei’s role in MEGA’s inception and early guidance and support makes it a big part of his enduring legacy.

**Fig. 3.**
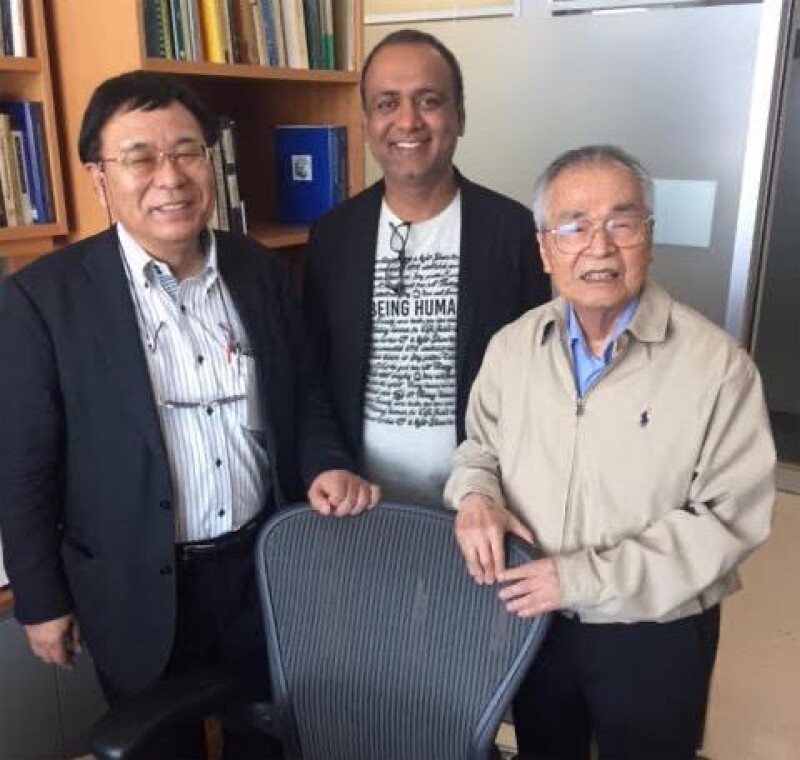
Masatoshi Nei (right) with Takashi Gojobori (left) and Sudhir Kumar (middle) in Dr Nei's office at Temple University (2017). Photo credit: Takashi Gojobori.

The MEGA project’s success appears to have inspired Dr Nei to write the book “*Molecular Evolution and Phylogenetics*,” published in 2000 with one of us (Sudhir Kumar; fig. 3). Portions of the MEGA manual served as precursors for chapters in the book, illustrating methods and concepts through worked-out examples and accompanied by downloadable data files and computer software packages from a webpage. This dedication to meeting the needs of practicing biologists contributed to Dr Nei amassing over 400,000 citations (Google Scholar, May 27, 2023) and earning recognition as one of the most highly cited scientists ever.

As a testament to his achievements, Dr Nei was elected a fellow of the American Academy of Arts and Sciences in 1990 and became a member of the US National Academy of Sciences in 1997. He also received numerous prestigious awards, including the International Prize for Biology in 2002, the Thomas Hunt Morgan Medal in 2006, the Kyoto Prize in Basic Sciences in 2013, and the John Scott Medal in 2017.

**Fig. 4.**
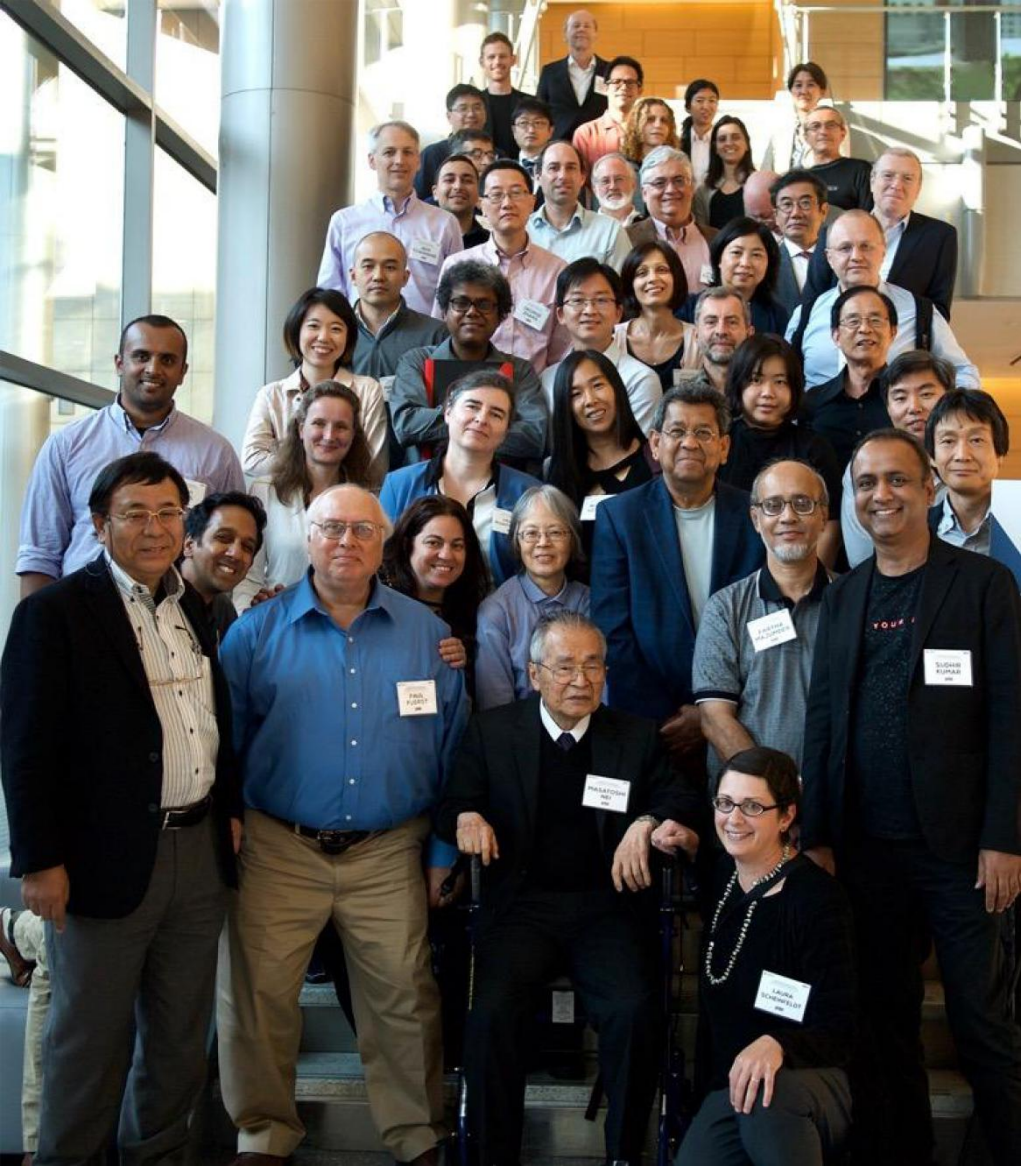
Masatoshi Nei (first row, middle, in a wheelchair) with his students and other attendees of the 2017 symposium celebrating his achievements at Temple University (photo credit: Sudhir Kumar).

Dr Nei’s pursuit to benefit biology and biologists through research and method development proved highly successful. He sought to design and develop biologically useful approaches that were easily understandable and relied on minimal assumptions. While he employed mathematical analysis to address biological questions, he remained uninterested in indulging in overly complex mathematical theories or purely theoretical methods. Dr Nei believed such pursuits were a waste of talent, as their assumptions often lacked biological realism and limited their practical applications. He expressed occasional dismay at the fascination of some brilliant scientists with complex mathematical and statistical models. As a result, he sought self-motivated students with interdisciplinary backgrounds, prioritizing those driven by a genuine interest in solving biological questions. His exceptional ability to identify such students is evident in the impressive list of doctoral (∼23) and postdoctoral (∼38) mentees he nurtured (fig. 4).

One example of Dr Nei’s dedication to simple methods is an article published in 2016 in collaboration with his last postdoctoral fellow. Amidst a global trend of using the most complex models and methods to construct phylogenetic trees from big data, he championed the virtues of molecular phylogenetics by emphasizing the use of the simplest measure of genetic distance—the proportion of bases that differ between sequences (*p* distance). Regardless of one’s stance on using *p* distance for building phylogenies, Dr Nei’s independent thinking, unswayed by “fashionable” trends in the field, commands admiration. Additionally, it is worth noting that the Hamming distance, an equivalent of *p* distance, is widely employed in machine learning and data science today.

Dr Nei’s independence and ingenuity were also evident in his support of the neutral theory. He was an early and fervent proponent of the neutral theory, demonstrating that it could explain many patterns of genetic variation and evolution when comparing expected and observed heterozygosities in various species in the 1980s. However, he felt that Kimura’s neutral theory could embrace negative selection even more. In his theory of mutation-driven evolution, Dr Nei proposed that statistical models incorporating negative selection can explain textbook examples of adaptive evolution, such as the evolutionary dynamics of protein variation resulting in sickle-shaped blood cells. According to his theory, in regions with high malaria prevalence, the advantage conferred by heterozygous genotypes came at the expense of negative selection of individuals with homozygous genotypes of mutant and wild-type alleles, as they would be prone to disease. This perspective aligned the observed patterns of sickle-cell polymorphism with negative selection and Dr Nei’s theory of mutation-driven evolution. Dr Nei felt that the term “natural selection” should stand for negative selection rather than positive selection. He extensively elaborated on his perspective in his final academic book, “*Mutation-Driven Evolution*” (2013). Interestingly, Dr Nei was not alone in this line of thinking, as Alfred Wallace, a contemporary of Charles Darwin and codiscoverer of evolution by natural selection, also proposed the “elimination of the unfit” as an alternative explanation to the popular “selection of the fittest” paradigm.

Beyond Dr Nei’s contributions to research articles and software applications, he played an instrumental role in establishing a platform for publishing research at the intersection of molecular biology and evolution. With Walter Fitch, he founded the journal *Molecular Biology and Evolution*, assisting numerous authors in improving their manuscripts and contributing to the journal’s popularity and high citation rate. Dr Nei also cofounded the Society of Molecular Biology and Evolution, which has become the premier organization for thousands of researchers interested in molecular evolution. These remarkable achievements firmly establish Dr Nei as a leading architect of the field.

In his autobiographical account, “*My Life as a Molecular Evolutionist*,” published in 2020, Dr Nei chronicles his personal and professional journeys, shedding light on the physical challenges he had to overcome. He lost his left eye in 1946 due to an accident while probing an ignition device of a bomb after World War II, resulting in difficulties in correctly judging distances, compounded by color blindness. It is truly remarkable that he invented many impactful measures of genetic distances, including one that bears his name. *One could say that he could see further than most of us!*

Dr Nei suffered a stroke in 2014, leading to his retirement from Pennsylvania State University and his move to New Jersey. He became an adjunct Laura H. Carnell Professor at Temple University in 2015 and began devoting more time to writing his memoir. Dr Nei passed away at the age of 92 on May 18, 2023. Posthumously, we discovered that Dr Nei derived enjoyment from listening to classical music and sculpting topiary. He is survived by his wife, two children, two grandchildren, four sisters, and many grateful academic offspring.

